# The curious case of a woman with two BRCA1 mutations in trans

**DOI:** 10.1186/1897-4287-13-S2-A11

**Published:** 2015-11-26

**Authors:** Rodney J Scott, Michelle Wong-Brown, Mary McPhillips, Susan Dooley, Allan Spigelman, Margaret Gleeson, Cliff Meldrum

**Affiliations:** 1 Discipline of Medical Genetics, Faculty of Health, University of Newcastle and Hunter Medical Research Institute, Newcastle, NSW, Australia

## 

Since the identification of *BRCA1 *twenty years ago there has not been a single unequivocal case of anyone (even in populations where there is a high frequency of founder mutations) harboring two *BRCA1 *mutations in trans, suggesting that this is embryonically lethal. These findings have been substantiated by animal models where no viable offspring *BRCA *null mice have been generated by crossing heterozygote carriers. It is known that Brca1 mice can be partially rescued by *Tp53 *mutations, but they do not survive well. Recently one patient has been reported with a nonsense mutation on one *BRCA1 *allele and a missense on the other. The patient in this study suffered from malignancy, intellectual disability and a number of other symptoms. The missense variant has not been extensively verified and as such cannot be verified as pathogenic at this time.

Here we report a woman, diagnosed with breast cancer during her fourth decade of life, who has been shown to harbour one nonsense mutation and a splice site mutation. Both changes results in the loss of wild-type *BRCA1*. The question raised by this finding is: How come this woman is alive and outside of her initial presentation is currently healthy? An in depth molecular analysis was performed which revealed the complexity of *BRCA1 *expression and how this could only be achieved by close examination of other family members. The outcome of the study represents the most likely explanation for this extraordinary case.

## Consent to publish

Written informed consent was obtained from the patient for publication of this abstract and any accompanying images.

